# Dimensions of Service Quality in Health-Fitness Clubs in China

**DOI:** 10.3390/ijerph182010567

**Published:** 2021-10-09

**Authors:** Karen Kaijuan Xu, Kenny Kuanchou Chen, Euisoo Kim, Jerónimo García-Fernández, John Nauright, James J. Zhang

**Affiliations:** 1 School of Economics and Management, Shanghai University of Sport, Shanghai 200438, China; 2 College of Sport Management, National Taiwan Sport University, Taoyuan 333, Taiwan; kcchen1205@gmail.com; 3 Department of Kinesiology, University of Georgia, Athens, GA 30602, USA; euisoo.kim25@uga.edu (E.K.); jamesz48@uga.edu (J.J.Z.); 4 Research Group of Management and Innovation in Sport Services, Leisure and Recreation (GISDOR), University of Seville, 41013 Seville, Spain; jeronimo@us.es; 5 Richard J. Bolte, Sr. School of Business, Mount St. Mary’s University, Emmitsburg, MD 21727, USA; profjohnnauright@gmail.com

**Keywords:** customer satisfaction, health-fitness clubs, Chinese market, service recovery

## Abstract

The purpose of this study was to explore the dimensions of service quality in fitness clubs in China and examine their impact on customer satisfaction. In Phase I of the study, we collected qualitative data from online comments related to service quality in 30 Tera Wellness clubs in Shanghai (k = 6252). Conducting content analysis, we synthesized the information and identified preliminary themes and formulated measurement statements. Phase II implemented a series of quantitative research procedures to examine the measurement properties of statements developed in Phase I. Conducting exploratory factor analysis, confirmatory factor analysis, and structural equation modeling analyses based on responses of club members (*N* = 533), we identified a total of 27 items in six dimensions: service recovery, service assurance, facility function, program operation, instructor quality, and staff performance. These factors significantly (*p* < 0.05) predicted customer satisfaction with fitness clubs in China. The findings highlight the importance of high-quality service delivery, service recovery, and service assurance and pinpoint specific areas for improvement.

## 1. Introduction

During the recent economic downturn, the Chinese government has chosen the sport industry as an important channel to boost business growth. Under a new economic policy, the fitness and leisure industry has gained recognition as “sun-rising” industry and received unprecedented support from the Chinese government. Consequently, the number of health-fitness enterprises has increased, including corporate fitness, fitness studios, 24-h gyms, and virtual fitness services. This fast-paced development has led to some growing pains; for instance, club managers regularly need to evaluate club operations in order to maintain or improve service quality and adapt to constantly changing business surroundings [1,2].

In the fitness industry, customer retention remains a key factor in success, and service quality continues to have a strong impact on that retention. Service quality in a fitness club directly relates to profit margin and overall club development in a competitive marketplace [3,4,5]. Effective management of service quality increases customer satisfaction, loyalty, and positive word-of-mouth [6,7]. Although several scholars have addressed the importance of service quality in managing fitness clubs (e.g., [4,8]), none has explored fitness service quality in the Chinese market. Previous findings suggest that no single model can measure service quality universally because clients from different cultural backgrounds are likely to have different expectations and needs [9,10]. As services expand globally, understanding how to measure service quality in different countries becomes increasingly important. Due to the political climate and cultural differences, Chinese customers might have different perceptions and expectations of fitness club service quality than Western customers [11].

China’s rapid rise to economic and political power has generated various social changes. For example, competition in China’s fitness industry has intensified. Recently, consumers have come to expect fitness clubs to be disciplined and honest and to expect service recovery and service assurance [12,13]. More generally, studies about service quality management in China feature descriptive statistics and fall short on reliability and validity assessment. Contextualized studies that target the dimensions of fitness club service quality and reveal variables that influence fitness club operations in China are crucial, especially in light of recent changes in Chinese economic conditions [12,14]. Some of these dimensions and variables are likely to overlap the ones that are relevant in Western fitness settings, while others are likely to differ in substantial ways.

The purpose of this study was to explore the dimensions of service quality in China’s fitness clubs and examine their impact on customer satisfaction. The findings should contribute to the literature by revealing variables that affect effective operation of fitness clubs in China. Some of those variables are likely to reflect sociocultural, political, and economic differences between China and the rest of the world. The findings should also help fitness club managers elevate service quality and better satisfy the needs and wants of Chinese consumers.

## 2. Review of Literature

In one of the earliest service quality studies, Parasuraman, Zeithaml, and Berry [15,16] proposed 10 criteria for evaluating service quality: credibility, safety, accessibility, communication, understanding of customers, reliability, sensitivity, ability, and etiquette. They categorized these criteria into five dimensions: tangibility, reliability, responsiveness, assurance, and empathy. Then, using exploratory factor analyses of retail organizations, they developed the SERVQUAL instrument. In the context of the current study, service quality in fitness clubs has two types, one based on the five generic dimensions of SERVQUAL and one that accounts for the unique characteristics, functions, features, and activities of health-fitness clubs [4,17,18].

Scholars have argued that SERVQUAL does not measure the specific operations of various service sectors. Health-fitness clubs, especially those in China, have unique features. For example, fitness clubs sell experiences, not goods. Personalized service plays a much more important role in fitness clubs today, given increasing market competition and shifting consumer demands [19]. Moreover, fitness clubs and consumers usually sign long-term contracts in China, anywhere from one to three years to life long. Given this significant commitment, consumers understandably consider as many factors as possible when selecting a club. These factors include, but are not limited to, distance to the club, equipment provided, atmosphere, staff, and cost of converting to other clubs [4,12]. The reality is that without an extraordinary reason (e.g., going abroad, pregnancy, job change, or accidental injury), fitness club consumers in China can rarely change to a different club [12]. Another important factor when promoting consumer loyalty is encouraging customers make an emotional investment in the club [20].

Constructs in a measure reflect the theoretical structure of a concept, which would be the case for service quality in health-related fitness clubs. Scholars have developed a number of measures to assess the service quality of fitness clubs in various settings. The Scale of Attributes of Fitness Services (SAFS) includes 30 items in five dimensions for evaluating service quality in Canadian fitness clubs [21]. The Center for Environmental and Recreation Management-Customer Service Quality (CERM-CSQ) model includes four factors: core service, staff quality, general facilities, and secondary service [22]. In a follow-up study, scholars condensed these four factors into three: employees, core service, and secondary services [23]. Kim and Kim [24] established the Quality Excellence of Sport Centers (QUESC) scale, which has 33 items for 11 factors of service quality in Korean sport centers: service environment, service staff attitude, service staff reliability, information provided, course items, personalized service, discounts, price, mood for relaxation, incentive, and convenience. Although the scale provides a wide spectrum of features, it might not be the most effective. Three factors (i.e., price, privilege, and incentive) contain only one item, and several other factors had an internal consistency coefficient lower than 0.70 [25]. These shortcomings emerged in a study by Papadimitriou and Karteliotis [26], who applied the QUESC model in the context of Greek sport and fitness clubs. Instead of an eleven-factor model, the findings suggested a four-factor model, which included instructor quality, facility function, program operation, and other services. The Scale of Service Quality for Recreation Sport (SSQRS) includes four factors: program quality, interaction quality, outcome quality, and physical environment quality [27]. SSQRS was adopted to assess perceived service quality of regular users of gyms in public sports centers in the UK, and physical environment perspectives (i.e., ambience and facility function) were found to be the most predictive of customer satisfaction, with equipment being generally more important than all other aspects (Polyakova & Ramchandani, 2020) [28]. Alexandris et al. [28] used catering, staff, water facilities, accessibility, dry facilities, and cleanliness factors to measure service quality in fitness clubs in England.

Lam et al. [4] established the Service Quality Assessment Scale (SQAS) model, which includes 40 items under 6 dimensions for evaluating service quality in U.S. fitness clubs, including staff quality, course project, dressing room, physical environment, fitness equipment, and child care area. The SQAS scale has been widely adopted in various research investigations into service quality issues in health-fitness clubs. García-Fernández et al. used facilities, employees, and programs factors to explore fitness center consumer loyalty and found that customers in private low-cost fitness centers put a greater weight in the quality of facilities and employees and customers in public fitness centers put a greater weight in programs. The authors suggested that fitness centers should conduct detailed analyses and monitoring of the provided service and take into consideration customer background characteristics when taking managerial actions to meet the needs and wants of customers [29]. According to Polyakova and Mirza, one criticism of Lam et al.’s scale is that it does not offer the measurement of overall perceived service quality and does not include an inter-client interaction aspect nor the outcome quality proposed by several other service quality models [30]. Leon-Quismondo conducted an importance–performance analysis of fitness center services quality while taking into consideration gender and age background of club members. The findings show that the level of congruence between importance and performance of fitness clubs was more evidently relevant to women than men. Female members emphasized more on core elements of club services, such as the variety and frequency of activity program offerings, personal training, quality of fitness equipment, and particularly provision of swimming pool and aqua-programs. Meanwhile, older club members put greater emphasis on the importance, performance, and their congruence in the areas of cleanliness of activity spaces and the safety of lockers than younger members [31]. These findings illustrate that no single model can capture service quality universally because clients with different cultural backgrounds are likely to have different expectations and needs [9,10,29]. 

Scholars have effectively evaluated service quality in fitness clubs in different countries and discovered that evaluation scales can differ substantially across cultures. Yu, Zhang, Kim, and Chen [8] used staff, program, locker room, physical facility, and workout facility factors to assess service quality in health-fitness clubs for senior consumers in Korea. In China, fitness club operations have been widely mediocre, unstandardized, and disorganized in a market with extensive competition [12,14]. However, the situation has begun to change with the recent policies implemented by the central government. Contemporary Chinese fitness consumers are better informed and have higher expectations; increasingly, they desire better and more standardized services from health-fitness clubs [30]. Building on previous findings, we explored the following themes to examine service quality in fitness clubs in China: service assurance, service recovery, facility function, program operation, instructor quality, and staff performance.

### 2.1. Service Assurance

The term “service assurance” refers to the ethical bottom line and code of conduct followed by clubs to minimize consumer doubts and risks during business activities. In China’s fitness industry, consumers now expect clubs to develop standardized and honest practices; they want psychological assurance and price assurance. Regarding psychological assurance, consumers often have to sign a contract that demands a personal commitment, but not any commitment on the part of the fitness club. Moreover, consumer information confidentiality has become an important indicator of service quality due to social media development. Psychological assurance also includes commitment to the consumers and follow-up treatment when employees leave and uncontrollable changes occur. The ability to make customers trust that fitness clubs have the means and the competence to deal with problems is a key part of psychological assurance. Price transparency is also critical to psychological balance. Uncertainty about price transparency, procedural fees (e.g., replenishment, card suspension, and membership cancellation), and reasonable rates have increased the perceived risk of losing money. Today, fitness club prices are largely open and transparent, and honest operation has become a consumer demand for currency security [30,31].

### 2.2. Service Recovery

The term “service recovery” refers to the process by which fitness clubs take action to make up for losses incurred through service mistakes in order to regain consumer trust. Service recovery management is a process of finding discrepancies and is an important source of information that clubs use to improve service quality. With the surge in consumer awareness of self-protection and rights protection, consumers expect clubs to take “emergency measures”, which exceed the standards of normal service. When a club does not fulfill its service pledge or properly resolve a customer complaint, a service error occurs [12,32,33]. Davidow [34] found that the speed of handling of customer complaints positively influenced word-of-mouth communication by customers. Thus, whether a club can quickly implement service correction directly affects perceived service recovery. 

### 2.3. Facility Function

The term “facility function” refers to the working condition of the main facilities and equipment that consumers use during workouts. High-quality equipment and supporting facilities attract new members, and subsequent maintenance is key to increasing member satisfaction. First, quantity and performance of new and old equipment encourage consumers to join specific clubs. In addition, consumer observations and comments indicate that post-maintenance and repair of facilities and equipment have caused widespread concern in consumers [4,17,35].

### 2.4. Program Operation

Fitness club trainers offer two types of programs: group and individual. Consumer judgments of program quality depend on curriculum setting and service delivery. The variety of programs and the novelty of program content attract consumer attention. The quality-of-service programs relate to the popularity and effectiveness of fitness clubs. In addition, fitness clubs face increasing competition from rapidly expanding boutique fitness studios and the emergence of tailor-made courses on the Internet. Convenient scheduling and control over course time, place, and attendance have become primary concerns of consumers [17,36].

### 2.5. Instructor Quality

Personal trainers provide the most direct and frequent service to consumers. More and more clubs use personal education programs as special services to increase their competitiveness. These programs attract members who seek pain relief, improved posture, rehabilitation, dietary support, enhanced fitness, and higher mental capacity through specific exercise. Members hire personal coaches and have the freedom to choose from a wide variety of personalities, teaching methods, and professional skills. Of chief importance to consumers are professional skills, the ability to build fitness confidence and maintain interest, personality, and degree of seriousness [4,21,36].

### 2.6. Staff Performance

General staff include the front desk and sales staff who make up fitness club payrolls. In today’s market, consumers pay an increasing amount of attention to the protection of their self-interests. For this reason, staff should conduct sales promotions (e.g., telephone, WeChat) in ways that neither disgust nor disrupt consumers from their normal fitness routines and daily work schedule. Fitness clubs should also consider offering the same services to new and existing members (e.g., group purchase, access to programs). In addition, most consumers expect to establish a long-term relationship with their club of choice, so employee honesty and reliability will directly affect perceived credibility of the entire club [32,37].

## 3. Method

We used a multi-method and multi-stage design, which is consistent with a psychometric approach [38]. In Phase I, we developed scale items designed to map the conceptual properties of service quality and identified items that would indicate service quality in China’s fitness clubs and have acceptable reliability. In Phase II, we statistically tested and confirmed the following: (a) measurement variables representing the service quality construct, (b) convergent and discriminant validity of the service quality dimensions, and (c) the concurrent validity and reliability of the service quality measure.

When designing the investigation procedures for the current study, we took into consideration that due to the rising of social media networks, many consumers would like express opinions via online forums, instead of going to express opinions directly to the company involved [39]. Whereas social networks can be used as an effective channel for marketing their products and services by organizations, they can also be used as the information source for third-party evaluations and even complaints as the networks help provide a platform for people to freely tell about their experiences, express their evaluative opinions, and communicate with their friends and even community connections at large [40]. Today, increasingly more government agencies, local authorities, and managers in organizations utilize information generated from social network communications in their decision making [41]. It is believed that gathering the details of social networks comments and use them to help make improvement in product delivery and service provision can help improve consumers’ satisfactions and in turn influence the overall success of enterprises [42]. For example, by analyzing online comments, Li assessed tourist satisfactions about the terracotta warriors scenic spot in Xi’an [43]. To study hotel consumer satisfaction, Xiong and Xu analyzed social network comments of hotel clients through conducted content analyses, which led to the formulation of a widely-adopted evaluation index system of hotel services [44].

### 3.1. Phase I

Sample and Data Collection. The health-fitness club is a profit-making organization that provide high-quality products, offer satisfactory customer services, and meet customer-centric goals [45]. The purpose of Phase I study was to explore the preliminary dimensions of service quality in China’s fitness clubs. 

Content analysis of reviews on Dazhong Dianping, a Chinese version of Yelp, helped us determine how Chinese customers evaluate service quality in fitness clubs. According to the Shanghai Sports Bureau website, there are 564 fitness clubs in Shanghai, including 225 independently operated facilities and 11 franchises. As franchise brands have regional systematic operation methods, we selected franchise brands that (a) operate more than three fitness clubs in Shanghai and (b) had at least 50 reviews in Dazhong Dianping. We identified 152 fitness clubs that satisfied those criteria (see Table 1). We then collected comments posted on Dazhong Dianping about these 152 fitness clubs between over a period of two years. The final sample included 6252 customer comments.

Coding Process. Using earlier posted comments, two coders identified themes, shared coding results, discussed categories, resolved conflicts, and decided on six themes that were consistent with existing theory and terminology: service recovery, service assurance, facility function, program operation, instructor quality, and staff performance [4,21,22]. To improve content validity, we had an expert panel examine the relevance, representativeness, and clarity of each theme. We asked five sport management professors who were regular fitness customers to examine independently each theme. Based on a standard of 80% agreement among the panel members, we retained all of the themes (see Table 1). 

We first conducted open-coding analysis using Nvivo 12. As each review typically contained more than one theme, the coders freely assigned each review into multiple categories when deemed necessary. For example, the following comment contained three different themes: “the traffic is so bad, there is only the 946 bus line; the bus comes every 20 min, the area is very small, the club is on the ground floor, and the air is so bad”. Coding yielded 13,369 thematic fragments, which we then categorized into different items. For instance, the example fit into three items: “traffic”, “area”, and “ventilation”. Consequently, we categorized all 13,369 fragments into different items, and decisions about item retention depended on frequency. We conducted a well-planned training session to make sure that the coders fully understood the codebook. Each coder randomly coded 600 reviews (i.e., 10% of the total sample). Krippendorf’s Alpha, which we used to test inter-coder reliability, ranged from 0.88 to 1.00. The final interrater reliability trial met the criteria of 80% agreement and 0.70 kappa for mutually exclusive items. We conducted descriptive statistical analyses using SPSS Version 23 and reported frequencies and percentages for all categories.

### 3.2. Phase II

Sample and Data Collection. The purpose of Phase II was to confirm and measure the dimensions of service quality by using the categories and themes derived from Phase I. Through a survey study, we conducted quantitative analyses to determine construct reliability and validity of the scale and to assess the impact of the dimensions on customer satisfaction. After approval from the Independent Ethics Committee at the university, we collected data from 30 randomly selected Tera Wellness clubs in Shanghai. Tera Wellness is the most well-known and biggest fitness franchise brand located in the various districts of Shanghai, with cumulative service of more than 1.0 million members and over 50,000 active members a day. The total number of Tera Wellness clubs was 58 in 2015, accounting for 25.7% of that franchise fitness clubs in Shanghai. As a part of the chain of clubs, these franchises are spread out throughout the city of Shanghai, in residential areas with different housing prices and residents of diverse income levels. 

After communicating with the CEO of Tera Wellness about the purpose of the study, we obtained permission to conduct the study from the CEO and from each of the branches. With the assistance from Tera Wellness, ten trained research assistants distributed the survey form to every tenth person entering the fitness clubs between July and September of 2015. Respondents first read informed consent forms, which promised anonymity and the right to refuse to participate. This process made sure that participation was voluntary. While a total of 600 individuals agreed to participate in the study, only 533 completed the survey form, representing a return rate of 88.8%. Of the 533 respondents, 246 (46.2%) were men, and 287 (53.8%) were women. A majority (89.83%) had at least a Bachelor’s degree, and the sample showed equal distribution across four age ranges: 19–25, 26–35, 36–45, and 46–55. Over half of them earned more than 10,000 yuan/month (52.7%). Most of them exercised 1–3 times a week (62.0%), and 56.5% had been a club member for 1–3 years, while 31.5% had been a club member for more than 3 years (see Table 2).

Measurement. We converted the categories, themes, and statements obtained in Phase I into the preliminary Scale for Fitness-Club Service (SFCS) that contained 40 survey items to assess the service quality factors. To test content validity, we asked five sport management professors specialized in fitness operation to examine the relevance, representativeness, and clarity of each item. Based on a standard of 80% agreement among the panel members [46], we retained all of the items, although we revised numerous items for clarity. To verify concurrent validity [38,47], we examined the service quality scale using an established service-related outcome variable: satisfaction [28,48,49]. We used the well-accepted three-item satisfaction scale developed by Mano and Oliver [50]. The final survey contained 40 items: (a) service recovery (7 items), (b) service assurance (8 items), (c) facility function (4 items), (d) program operation (4 items), (e) instructor quality (4 items), (f) staff performance (5 items), (g) satisfaction (6 items), and (h) demographic information (5 items). We measured all items on a 5-point Likert-type scale (1 = strongly disagree; 5 = strongly agree) and used mean values for each variable. The questionnaire was originally in English, so we had two Chinese-English bilingual scholars translate the 42 items from English to Mandarin for the respondents living in Shanghai. The first author then translated the Chinese questionnaires into English, yielding some minor word changes.

Data Analyses. We randomly split the sample into two sub-samples (266 respondents for exploratory factor analysis [EFA], 265 for confirmatory factor analysis [CFA]). We detected no missing data. First, EFA using 40 items determined the reliability and validity of the service quality scale. We implemented principal component analysis with varimax rotation. CFA using 265 samples and AMOS 23 further validated the latent structure. Structural equation modeling (SEM) established that the six factors of service quality had an impact on satisfaction. We used a number of goodness-of-fit statistics to evaluate the measurement model: chi-squared to the degrees of freedom ratio (1 < χ^2^/df < 3), root mean square error of approximation (RMSEA < 0.08), and comparative fit index (CFI > 0.90) [51]. Furthermore, to verify concurrent validity, we followed a two-step procedure using AMOS 23 for all 533 samples. CFA indicated that the measurement model was sufficient.

## 4. Results

We performed a frequency analysis of customer reviews to figure out the main categories of service quality in fitness clubs. As shown in Table 3, the two most popular categories were facility function (23.16%) and staff performance (22.07%), which were roughly equivalent. Regarding facility function, 10.81% of the reviews concerned “performance of equipment”, indicating that customers cared about equipment quality the most, and 7.12% concerned “supporting facilities in good condition”. Regarding staff performance, 9.84% of the reviews concerned “respect members’ needs”, and 8.75% concerned “moderate promotion”. Following facility function and staff performance, around 18.03% reviews mentioned instructor quality. Service assurance (15.86%) garnered slightly more responses than service recovery (12.43%), corroborating our suggestion that these two new categories are important service quality measures for China’s fitness clubs. Regarding service assurance, 3.27% reported “confidential information”, and 2.19% reported “practice verbal promise”. Regarding service recovery, 3.37% reported “solve problems within promised time”, and 1.86% reported “skill to handle complaint”. Program operation was the least mentioned category (8.46%). Table 3 presents the distribution of categories and the themes in each category.

To identify SFCS dimensions of service quality in fitness clubs in China, we first conducted EFA using 32 items from Phase I, utilizing principal component analysis and VARIMAX rotation. We found a five-factor solution explaining 75.26% of the variance. We adopted two metrics to determine factor retention and the respective items: (a) factors with an eigenvalue greater than or equal to 1.0 and (b) items with a factor loader greater than or equal to 0.40. The results revealed an eight-factor solution explaining 67.45% of the variance. We removed five items with cross-loadings on more than one factor [47]. Afterward, analysis revealed 27 items in six dimensions, with 66.6% of the variance explained: Service Recovery (7 items), Service Assurance (6 items), Facility Function (4 items), Program Operation (4 items), Instructor Quality (4 items), and Staff Performance (3 items). The second EFA achieved a more parsimonious scale. We named the six dimensions based on the content of the items within each one (see Table 4).

**Figure 1:**
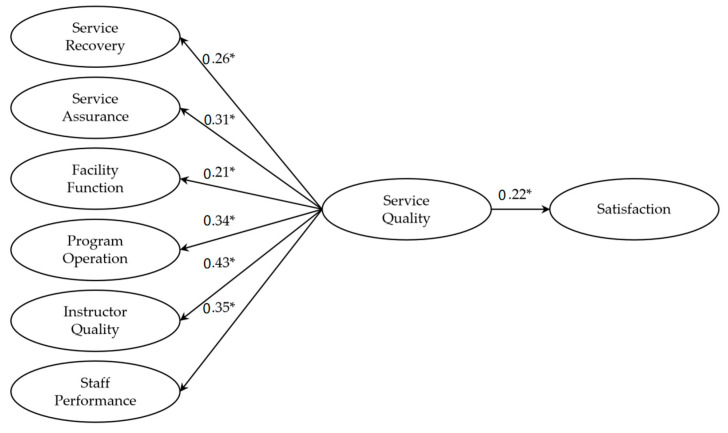
Research Model Resulted from Structural Equation Model Analysis. Note. * *p* < 0.01.

We conducted CFA by using the 265-case sample and AMOS 23 to test the reliability and validity of the new service quality scale. In the first CFA iteration, the model showed marginal fit (χ = 717.53, χ^2^/df = 2.14; RMSEA = 0.05; CFI = 0.91). The factor loadings for all 25 items were between 0.62 and 0.83, and the composite reliability was between 0.77 and 0.89, showing satisfactory reliability of the scale [51]. Convergent validity was sufficient, based on factor loadings (r > 0.40) and average variance extracted (AVE > 0.50) [47,51] (see Table 5). Discriminant validity of the scale was also sufficient; AVE exceeded the squared correlations for all factors. Therefore, the service quality scale was acceptable for further examination. To verify concurrent validity, we examined the service quality scale using an established outcome variable of satisfaction. CFA verified the convergent and discriminant validity of the service quality factors. The solution indicated acceptable model fit (χ^2^/df = 2.78; RMSEA = 0.06; CFI = 0.90). The composite reliability scores were 0.71–0.89, with AVE between 0.60 and 0.88, greater than the corresponding squared correlations for all service quality dimensions and satisfaction, confirming reliability and convergent and discriminant validity. SEM revealed that, in terms of the impact of the six factors of service quality on satisfaction, the model showed marginal fit (χ^2^/df = 2.92; RMSEA = 0.06; CFI = 0.89) (see Figure 1). All path weights among the six factors of service quality, as well as satisfaction, were significant (*p* < 0.01) (see Table 6 and Figure 1).

## 5. Discussion

In an effort to help managers of fitness clubs in China solve contemporary problems, we formulated a measurement model of service quality based on previous research findings and content analysis of 6252 customer reviews of fitness-club services in Shanghai. In particular, we identified a number of new items that were not a part of similar scales developed in Western studies. Following rigorous investigation, our Scale for Fitness-Club Service (SFCS) exhibited sound measurement properties for six factors: Service Recovery, Service Assurance, Facility Function, Program Operation, Instructor Quality, and Staff Performance. This measurement model, containing factors that predict customer satisfaction, has profound implications for future research about fitness club services and managerial practices in China.

The theoretical contribution of this study is a new, innovative, and psychometrically sound measure of service quality for health-fitness clubs. Most researchers agree that facilities, programs, and staff are crucial to fitness club operation [24,26,30,31,52] because they are just foundational and critical [4]. Previous service quality measurement models (e.g., SERVQUAL, CERM-CSQ, SAFS, QUESC, SQAS) have all contained facilities, instructors, and programs as critical elements that attract new fitness club members and help retain existing members. In the current study, the identified factors of Facility Function, Program Operation, Instructor Quality, and Staff Performance in the resulted SFCS scale and the related findings of these factors being predictive of consumer satisfaction have confirmed and even further reinforced their relevance and importance that were found in previous research findings. Even so, while these findings are widely considered applicable to many fitness club settings throughout various parts of the world, they have become somewhat inadequate with today’s consumer demands and expectations. The scope of examining service quality of fitness-clubs is continuously evolving and needs to be adjusted with the changing social environment over time. Compared to consumer knowledge in the past two decades, today’s fitness club members are better informed of consumer rights, more aware of industrial standards of practice, more savvy about their consumption options in part due to the convent information sources made available by the internet and digital technology, more communicating with other consumers due to the available platforms of social media networking, and more willing to switch to another service in a highly competitive marketplace when their needs and wants are not satisfied. Thus, it is not surprising that two new factors, Service Assurance and Service Recovery, emerged in the findings of this study, which were grounded in comments made voluntarily by consumers via social media network and further confirmed through a rigorous deductive reasoning process that included exploratory and confirmatory factor analyses and structural relationship examination. Findings of these two factors are major contributions of this study to the existent literature that have already revealed the likely presence of these perspectives. What is absent is the lack of sufficient empirical evidence [12,30,31,32,33]. The findings of this study have helped fill the void in this regard.

The findings of this study revealed that safe, high-quality equipment and state-of-art facilities (e.g., shower, toilet, locker room, pool) [4,28,30,53] continue to be major considerations by fitness club members when selecting and staying as a member of a club. It needs to be highlighted that although consumer attention and demand may be evolving over time, their expectations for the basic functions of a fitness club remain stable. For club managers, high-quality equipment and facilities are some of the fundamental elements when recruiting and sustaining club members. To meet the diverse needs of members, group programs need to be well formulated, offered, and delivered [19,24,26,28]. Most of the items in this factor are included in previous studies, such as the variety of programs and the rationalization of program arrangements and scheduling [4,24]. Nonetheless, there is one new item found for this factor in the current study, which represents program innovation that is meant for improving customer experience with virtual interactions and online communications. One explanation is that in health-fitness clubs today, as in many other areas of community services and business operations, high-tech and internet services are becoming a default consumer demand [54]. Thus, the addition of this item is just a matter of keeping up with time. For a fitness club, regular technology upgrading is an inevitable reality. 

Naturally, many fitness clubs regard program trainers and instructors’ education, knowledge, skills as the core elements for a club’s market competitiveness. Customization of instructions and trainings is becoming a trendy practice nowadays [55]. Managers of fitness clubs need to be particularly cognizant that today, their club members are particularly observing about professional qualification, knowledge foundation, and skill sets of the personnel working in the club. Through good communications and motivations, these trained professionals are expected to help individual members achieve their personal fitness and well-being goals. Besides a trainer or instructor’s professional competence, his/her professional characteristics in terms of responsibility, reliability, and accountability are viewed critical by club members [26]. It is also necessary to note that numerous researchers have reported that in recent years, there have been significant changes in customer expectations about supporting staff’s performance in fitness clubs [18,56]. While expectations for empathy and friendliness are receiving less emphasis from consumers, their desires for fulfilling personal interests and achieving individual goals are increasingly becoming forefront. Very importantly, they do not want to be disrupted during their normal exercise routines, not even in daily lives, which highlights the importance of choosing appropriate channels and adopting appropriate schedules of communicating with current and potential club members, especially when doing club promotions. 

Findings related to the two new factors of Service Assurance and Service Recovery and including them in the newly developed SFCS are original and significant. According to Anderson et al., malpractices or ill-intentioned practices in some mainstream businesses and fitness clubs in specific have led to distrust among consumers to some extent. Some of the managerial issues and consumer concerns are likely caused by such incidents as breach of personal information, lack of fulfillment of contract obligations and promises, price scalping, and inadequate safety and security procedures. When consumer rights are violated, membership benefits are not delivered, promises are not materialized, or people feel cheated, they are just unlikely renew their membership; on the contrary, they are more likely inform their dissatisfaction to their family, friends, and community connections via social media networks [52]. As the fitness industry in China is relatively a new development when compared to those in Western countries, some of these problems are comparatively more prevalent. Industry standards need to be developed; in the meantime, providing, practicing, and ensuring service assurances, such as completely honoring a member’s contract, maintaining a reasonable and stable membership price, avoiding any hidden agenda and price scalping, and preserving facility placement and spacing for safety reasons, are extremely important for earning consumer trust, loyalty, and membership retention. According to Lam et al. [4] and Yu et al. [8], club members know when they are treated with professionalism, honesty, care, and genuine interest. Chinese customers expect inclusion and reinforcement of professional ethic codes, which would help ease people from developing suspicions and worries. As a market-oriented entity, a fitness club should comply with basic business norms, implement corporate social responsibility, and ensure individual confidentiality [7].

It is never desirable to make mistakes in fitness club operations. Yet, errors do occur. When a club does not fulfill its professional obligations or when a club does not resolve a customer complaint in a timely and satisfactory fashion, a service error occurs and remains unresolved [12,32,33]. The effectiveness and efficiency of resolving customer complaints positively influenced an individual’s word-of-mouth communication and ultimately, his/her membership loyalty [34]. Handling complaints of customers can be an opportunity to communicate with customers, make up for mistakes, and enhance mutual trust between customers and club managements [10,49,52]. Effective service recovery can eliminate consumer dissatisfaction and help consumers rebuild loyalty after service failure [32,39,57]. Fitness clubs should develop standard “contingency packages” and “emergency procedures”, as recommended by Afthinos et al. [39]. It is necessary to keep in mind that customers evaluate service recovery behavior from outcome fairness, process fairness, and interaction fairness when fitness clubs are dealing with customer complaints [58,59,60]. Employees should go through formal training to master emergency skills. They need to be aware of standard protocols when encountering membership complaints, develop professional skills to handle complaints, sincerely communicate about procedures and timelines of problem solving to the member(s) involved, and seek for effective remedial solutions.

Our service quality scale, SFCS, can be adopted to assist with identifying the impact of fitness club interactions between customers and service providers on personal, organizational, and collaborative outcomes [7,61,62]. Overall, our findings contribute to a conceptual and empirical understanding of service quality within the fitness industry, revealing the axiological effects that service quality has on service outcomes. The findings support the premise that service quality is multi-dimensional. Although facility function, program operation, instructor quality, and staff performance are common factors in most existing studies, our measure is the first to include service recovery and service assurance, applied specifically to the Chinese fitness industry [37,63,64]. Thus, our scale provides a unique understanding of how consumers perceive service initiatives and how those initiatives elicit favorable consumer evaluation.

Our findings have some practical significance, as well. The new measure should help fitness professionals appraise service effectiveness and measure progress toward high service quality. Given that the six factors in the model emerged from customer comments, fitness club managers should pay close attention. However, flawless service is a strenuous task. Club managers should study their unique situation to determine how much more attention the three significant factors (i.e., Service Assurance, Service Recovery, Facility Function) need. The findings suggest that customers expect clubs to be consistent with basic ethical norms, psychological contracts, and social responsibility. Clubs must further comply with mandatory regulations and certification procedures (e.g., relevant laws and regulations, standards, and safety guidelines), and managers should make a particular effort to enhance security services in order to achieve customer satisfaction in China. Furthermore, the findings shed light on the positive impact of service quality on satisfaction [17,31,65,66]. Service quality, when valued positively, can increase a service provider’s competitive advantage. Thus, industry professionals should consider increasing service quality, especially in the domains of service assurance, service recovery, and facility function [12,67].

## 6. Conclusions

The service quality scale developed in the current study has sound psychometric properties and can measure service quality in fitness clubs in China. The factors include Facility Function, Program Operation, Instructor Quality, Staff Performance, Service Recovery, and Service Assurance. The first four factors and their items reflect basic requirements and norms expected by consumers across the fitness industry. The other two factors (i.e., Service Recovery and Service Assurance) reflect new changes in consumer demand. Conceptually, the first four factors and items, while reflecting perceived benefits, have changed little over time, whereas the other two factors and items, which relate to perceived cost, have undergone significant change. From a customer perspective, the first four factors indicate expectations for personal service, and the other two factors indicate confidence in the standardization of fitness club services. Our findings suggest the possibility of integrating standardization and customization into a single framework. 

Given ever-changing customer expectations and the expansion of competitive market environment in China and even globally, the meaning of service quality will continue to evolve. Our study was based on qualitative and quantitative data collected in 2014; although the resulted research evidence remains contemporarily relevant and to some extent, forward-thinking, changes in market environment and advances in technology in the past few years are apparent and potentially influential of health-fitness club operations. In particular, new technologies in online instructions, streaming service, exercise apps, home exercise equipment, and even healthcare and insurance policies have continued to transform consumer needs and drive changes in the fitness industry [55,68]. Starting in late 2019, the impacts of the COVID-19 pandemic on all aspects of human life around the world, both immediate and repercussive, cannot be overstated. This unprecedented virus and its aggressiveness have substantially altered ways how people carry out their daily lives and how organizations have to adapt their business operations. While the severity of this plaguing disease reminds people of the importance and necessity of doing exercise and participating in sports to help build up individual immune systems, it puts added requirements on the management teams of health fitness clubs [37,69]. Under the very difficult circumstance, health fitness clubs are expected to closely follow governmental, local, and healthcare agency’s policies and regulations to prevent the spread of the disease and protect the wellbeing of their members who choose to use the facilities. Some of the notable changes may include, but are not limited to the following: (a) increased spacing requirement between equipment, facilities, and individuals, (b) installing sanitized ventilation system, (c) frequently sanitizing and cleaning the facilities and equipment, (d) making it convenient for people to access sanitizing materials, (e) offering smaller group or even individual instructional programs, (f) offering online, live instructional or training programs, (g) converting indoor activities to outdoors, (h) developing outdoor and adventure fitness activity programs, (i) working with local health authorities to offer testing and vaccination services, and (j) developing therapeutic exercise programs to help those who have been recovering from suffering from the disease. As the data collection of this study was completed prior to the beginning of the COVID-19 pandemic, the feasibility and viability of these prevention procedures and alternated services were not taken into consideration in the current study. Surely, these speculations and suggestions need to be empirically examined in future studies, both in China and beyond. Regardless, health-fitness clubs should make better preparations and develop effective response protocols for future unpredictability and crisis management.

Finally, it is necessary to point out that the data for the current study were collected through members of 30 fitness clubs. These clubs were franchises under one business corporation, namely Tera Wellness. Although these franchises were located in different parts of the city of Shanghai and were positioned to serve clienteles of different sociodemographic backgrounds, they were under the management principles and operational procedures of the same corporation. The responses of club members to the survey administration could represent homogeneous perceptions and opinions of consumers. Future studies should consider including health-fitness club members representing diverse corporations, brands, and settings to enhance the generalizability of the research findings. Similar investigations are also suggested to be conducted in Chinese cities beyond Shanghai as the levels of economic and industrial advancement of Shanghai are rather ahead of other regions and cities in China, which could lead to differences in residents’ lifestyles and participations in health-fitness activities. When possible, cross-validations in other parts of the world would be very meaningful for the topic area of the current study.

## Figures and Tables

**Table 1:** Survey Samples in Dazhong Dianping in China.

Samples Name	Total Number of Fitness Clubs	Selected Number of Fitness Clubs in This Study	Selected Number of Reviews
Sample 1	3	3	74
Sample 2	8	8	357
Sample 3	39	21	607
Sample 4	3	3	140
Sample 5	5	5	151
Sample 6	10	8	1492
Sample 7	7	6	253
Sample 8	34	29	1460
Sample 9	16	16	318
Sample 10	5	5	175
Sample 11	58	48	1225
Total	188	152	6252

**Table 2:** Sociodemographic Characteristics of Research Participants (*N* = 533).

Variable	Category	*n*	%
Gender	Male	249	46.8
	Female	284	53.2
Academic	Senior high school/vocational school/technical school and below	49	9.1
	Undergraduate Education	380	71.3
	Graduate Degree	104	19.6
Career	Temporarily unemployed (including students; stay-at-home; retired)	89	16.7
	Freelance (including self-employed)	100	18.7
	Employees of enterprises and public institutions	255	47.9
	Management of enterprises and institutions	89	16.7
Age	18 years or younger	14	2.6
	19 to 25 years old	115	21.6
	26 to 35 years old	270	50.7
	36 to 45 years old	78	14.6
	46 to 55 years old	40	7.5
	56 years or older	16	3
Monthly income	6000 yuans or less	125	23.5
	6001 to 8000 yuans	115	21.6
	8001 to 12,000 yuans	115	21.6
	12,001 to 15,000 yuans	48	9.0
	15,001 to 30,000 yuan	84	15.8
	More than 30,000 yuans	46	8.6

**Table 3:** Coding Categories and Themes Derived from the Content Analyses (9147 items).

Category	Theme Classification	*N*	%	% of Stores
Service Recovery	Skill to handle complaint	170	1.86	64%
	Simplicity of procedure when handle complaint	135	1.48	63%
	Announce progress of handle complaint	155	1.69	67%
	Effectiveness of remedial measures	102	1.12	73%
	Responsibility taken	137	1.50	63%
	Quickly handle complain	299	3.27	66%
	Solve problems within promised time	139	1.52	67%
	Sum	1137	12.43	
Service Assurance	Fulfillment contract	126	1.38	69%
	Confidential Information	290	3.17	94%
	Practice Verbal promise	200	2.19	71%
	Stability price	138	1.51	54%
	Reasonable price	137	1.50	60%
	Facility placement and spacing	152	1.66	56%
	Provision and implementation of emergency measures	100	1.09	49%
	Suitability of exercise numbers	308	3.37	95%
	Sum	1451	15.86	
Facility Function	Performance of equipment	989	10.81	95%
	Up-to-date equipment	245	2.68	61%
	Supporting facilities in good condition	651	7.12	89%
	Total number of equipment	233	2.55	71%
	Sum	2118	23.16	
Program Operation	Variety of programs	205	2.24	71%
	Professional of program	230	2.51	71%
	Program arrangement	189	2.07	55%
	Program reservation	150	1.64	57%
	Sum	774	8.46	
Staff Performance	Respect members’ needs	900	9.84	96%
	Moderate promotion	800	8.75	81%
	Consistent services	188	2.06	52%
	Reliable staff	131	1.43	61%
	Respect members’ needs	900	18.02	96%
	Sum	2019	22.07	
Instructor Quality	Professional skills of instructor	568	6.21	78%
	Knowledgeable guidance	124	1.36	45%
	Reliable instructor	456	4.99	45%
	Decent behavior	500	5.47	88%
	Moderate promotion	400	4.37	66%
	Sum	1524	18.02	

**Table 4:** Exploratory Factor Analysis with Maximum Likelihood Extraction and Varimax Rotation.

Factor/Item	SR	SA	FF	PO	IQ	SP
Skill to handle complaint	0.815	0.142	0.119	0.084	0.068	0.179
Simplicity of procedure when handle complaint	0.783	0.202	0.029	0.154	0.132	0.019
Announce progress of handle complaint	0.779	0.212	0.159	0.115	0.035	0.156
Effectiveness of remedial measures	0.749	0.162	0.155	0.105	0.106	0.222
Responsibility taken	0.713	0.228	0.073	0.277	0.029	−0.006
Quickly handle complain	0.708	0.314	0.085	0.169	0.099	−0.018
Solve problems within promised time	0.517	0.346	0.241	0.058	0.153	0.201
Practice Verbal promise	0.227	0.796	0.121	0.010	0.119	0.103
Fulfillment contract	0.220	0.737	0.050	0.179	0.119	0.196
Stability price	0.261	0.732	0.126	0.126	0.045	0.164
Confidential Information	0.278	0.728	0.084	0.119	0.216	0.040
Reasonable price	0.165	0.625	0.025	0.116	0.213	0.147
Facility placement and spacing	0.197	0.605	0.187	0.254	0.131	0.171
Up-to-date equipment	0.069	0.101	0.823	0.196	−0.008	0.056
Performance of Equipment	0.057	0.124	0.765	0.318	0.215	−0.020
Total number of equipment	0.202	0.175	0.715	−0.009	0.161	0.076
Supporting facilities in good condition	0.233	0.025	0.675	0.218	0.153	0.197
Program reservation	0.136	0.065	0.142	0.775	0.081	0.143
Variety of program	0.169	0.173	0.098	0.753	0.083	0.124
Program arrangement	0.228	0.155	0.254	0.679	0.211	0.055
Outcome of program	0.185	0.214	0.218	0.609	0.100	0.170
Professional skills of instructor	0.127	0.218	0.154	0.118	0.822	0.182
Reliable instructor	0.125	0.193	0.078	0.293	0.780	0.113
Knowledgeable guidance	0.116	0.234	0.246	0.041	0.766	0.136
Moderate promotion	0.121	0.201	0.059	0.076	0.130	0.798
Respect members’ needs	0.165	0.140	0.097	0.193	0.151	0.754
Consistent services	0.151	0.349	0.127	0.228	0.135	0.617

Note. SR = service recovery; SA = service assurance; FF = facility function; PO = program operation; IQ = instructor quality; SP = staff performance.

**Table 5:** Cronbach’s Alpha, CR, and AVE Coefficients of the Retained SFCS Factors.

Factor	Item	CR	AVE	Alpha	Cronbach’s α
Service Quality Service Recovery	7	0.887	0.532	0.914	
Service Assurance	6	0.856	0.500	0.866	
Facility Function	4	0.834	0.557	0.796	0.837
Program Operation	4	0.799	0.500	0.816	
Instructor Quality	3	0.832	0.624	0.856	
Staff Performance	3	0.769	0.529	0.784	
Service Satisfaction	3	0.713	0.881	0.701	0.701

**Table 6:** Discrimination Validity of the Retained SFCS Factors.

Factor	Service Recovery	Service Assurance	Facility Function	Program Operation	Instructor Quality	Staff Performance	Service Satisfaction
Service Recovery	0.729						
Service Assurance	0.582	0.707					
Facility Function	0.346	0.365	0.746				
Program Operation	0.470	0.483	0.511	0.707			
Instructor Quality	0.371	0.512	0.425	0.453	0.790		
Staff Performance	0488	0.552	0.325	0.415	0.531	0.727	
Service Satisfaction	0.563	0.611	0.514	0.509	0.484	0.430	0.939

Note: Values on the diagonal represent the square root of each variable AVE, and the values on the off-diagonal are the correlation coefficient between the factors.

## Data Availability

Data for the current study are available upon request.
